# Detection of influenza A and B with the Alere™ i Influenza A & B: a novel isothermal nucleic acid amplification assay

**DOI:** 10.1111/irv.12303

**Published:** 2015-02-27

**Authors:** Briony Hazelton, Timothy Gray, Jennifer Ho, V Mala Ratnamohan, Dominic E Dwyer, Jen Kok

**Affiliations:** a Centre for Infectious Diseases and Microbiology Laboratory Services, Institute for Clinical Pathology and Medical Research, Westmead HospitalWestmead, NSW, Australia; b Westmead Clinical School, University of Sydney, Westmead HospitalWestmead, NSW, Australia; c Marie Bashir Institute for Infectious Diseases and Biosecurity, University of Sydney, Westmead HospitalWestmead, NSW, Australia; d Centre for Research Excellence in Critical Infection, University of Sydney, Westmead HospitalWestmead, NSW, Australia

**Keywords:** Influenza, isothermal nucleic acid amplification, rapid diagnostic test(s), sensitivity, specificity

## Abstract

**Background:**

Rapid influenza diagnostic tests (RIDTs) have an important role in clinical decision-making; however, the performances of currently available assays vary widely.

**Objectives:**

We evaluated the performance of the Alere™ i Influenza A&B (Alere™ iNAT), a rapid isothermal nucleic acid amplification assay that has recently received FDA clearance, for the detection of influenza A and B viruses during the Australian influenza season of 2013. Results were compared to two other RIDTs tested in parallel; Quidel Sofia® Influenza A+B fluorescent immunoassay (FIA) and Alere™ BinaxNOW® Influenza A & B immunochromatographic (ICT) assay.

**Methods:**

A total of 202 paired nasopharyngeal swabs collected from patients ≥16 years old with an influenza-like illness (ILI) were eluted in 2 ml of universal transport medium (UTM) that was used to perform all three RIDTs in parallel. Reverse-transcription polymerase chain reaction (RT-PCR) was used as the reference standard.

**Results:**

Compared to RT-PCR, Alere™ iNAT detected 77·8% influenza A positive samples versus 71·4% and 44·4% for the Quidel Sofia® Influenza A+B FIA and BinaxNOW® Influenza A & B ICT assay, respectively. For influenza B, Alere™ iNAT detected 75% of those positive by RT-PCR, versus 33·3% and 25·0% for Sofia® and BinaxNOW®, respectively. The specificity of Alere™ iNAT was 100% for influenza A and 99% for influenza B.

**Conclusions:**

Alere™ i Influenza A&B is a promising new rapid influenza diagnostic assay with potential point-of-care applications.

## Background

Rapid influenza diagnostic tests (RIDTs) have an important role in clinical decision-making as a rapid diagnosis of influenza A or B can facilitate the prescription of antivirals, reduce unnecessary pathology testing and antibacterial therapy and allow the implementation of appropriate infection control and public health measures.^1^,^2^ Currently, available RIDTs such as lateral flow or fluorescent immunochromatographic assays detect the presence of influenza A and B virus nucleoproteins. Although these assays are relatively simple to perform and can provide results within 10–30 minutes, they suffer from inaccuracies with widely disparate published sensitivities and specificities.^3^,^4^ Their performance is affected by various factors including patient age, duration of illness before sample collection, sample type and circulating influenza virus subtypes.^4^,^5^

Alere™ i Influenza A & B (hereafter Alere iNAT; Alere Scarborough, Inc., Scarborough, ME, USA) is a novel RIDT that utilises isothermal nucleic acid amplification for qualitative detection of influenza A and B viruses. The technical details of Alere iNAT have been recently described, but in brief, the assay involves real-time fluorescence-based detection of short amplicons following exponential isothermal amplification.^6^,^7^ Unlike common nucleic acid amplification assays that require nucleic acid extraction followed by thermal cycling using specialised equipment, Alere iNAT is a partially automated process that can be completed in <15 minutes using a small, bench top footprint instrument. Isothermal nucleic acid amplification has been recently demonstrated as an accurate diagnostic platform for the detection of influenza viruses; however, previously available assays have turnaround times of over an hour.^8^,^9^

## Objectives

Using real-time reverse-transcription polymerase chain reaction (RT-PCR) as the reference standard, Alere iNAT was evaluated for the detection of influenza A and B viruses during the Australian influenza season of 2013, where cocirculation of influenza A(H1N1)pdm09, seasonal A/H3N2 and influenza B was observed.^10^ Results were compared to two other RIDTs tested in parallel; Quidel Sofia® Influenza A+B fluorescent immunoassay (FIA) (hereafter Sofia; Quidel Corp., San Diego, CA, USA) and Alere™ BinaxNOW® Influenza A & B immunochromatographic (ICT) assay (hereafter BinaxNOW; Alere Scarborough, Inc., Scarborough, ME, USA).

## Study design

Paired Dacron® or flocked nasopharyngeal swabs collected from patients ≥16 years old with an influenza-like illness (ILI) were transported to the laboratory in viral or universal transport media (UTM™; Copan Italia, Brescia, Italy). UTM or swab tips transported in viral transport media were diluted in UTM to give a final volume of 2 ml, vortexed for 10 seconds and then divided into two aliquots; one for RT-PCR and the other for testing with RIDTs. All samples were refrigerated between 2 and 8°C if immediate testing was not possible and processed within 24 hours of collection.

Alere iNAT, Sofia and BinaxNOW were performed as per the manufacturers’ instructions.^11^–^13^ For Alere iNAT, the supplied sample receiver (containing elution buffer) and test base (containing lyophilised nucleic acid probes, internal control and fluorescent molecular beacon) were placed into the Alere i instrument. After initialisation, 200 μl of UTM was added to the sample receiver and then transferred via the included transfer cartridge to the test base, initiating target amplification. The instrument provided sample and reagent heating, agitation and target detection, and an objective qualitative report at the end of amplification.

Nucleic acid extraction for RT-PCR was performed on 200 μl of UTM from each sample using the Qiagen BioROBOT EZ1 instrument (Qiagen, Valencia, CA, USA) with an elution volume of 60 μl. Multiplex amplification was performed using the Roche LC 480 real-time instrument (Roche Diagnostics GmbH, Mannheim, Germany). The assay targets the influenza B virus nucleoprotein and matrix protein for all influenza A virus subtypes, including influenza A/H1N1, influenza A(H1N1)pdm09 and influenza A/H3N2, as previously described.^5^ Other respiratory viruses detected by this multiplex assay include rhinovirus, enterovirus, respiratory syncytial virus (RSV), parainfluenza viruses 1–3, human metapneumovirus (hMPV) and human adenovirus.

Where any of the RIDTs were positive for influenza B and RT-PCR was negative, the RT-PCR result was confirmed by testing with two different primer pairs. A published conventional thermal cycling method was used for the haemagglutinin gene primers with amplicons analysed by gel electrophoresis.^14^ A one step real-time RT-PCR was performed for matrix gene detection (CDC Influenza Division, Atlanta, GA, USA). Concordance of these two assays was taken to represent the true result.

## Results

Forty-eight (23·8%) samples collected from 202 adults (median age 56 years, range 16–94 years) were positive for influenza virus by RT-PCR, 36 (17·8%) influenza A and 12 (5·9%) influenza B. Twenty-eight (77·8%) of the influenza A positive samples were subtyped as A(H1N1)pdm09, 7 (19·4%) as A/H3N2, and co-infection with A(H1N1)pdm09 and A/H3N2 was identified in one (2·8%) sample. In addition, 34 (16·8%) samples were positive for other respiratory viruses including rhinovirus [*n* = 11 (5·4%)], hMPV [*n* = 11 (5·4%)], RSV [*n* = 7 (3·5%)] and human parainfluenza virus 3 [*n* = 5 (2·5%)]. The Alere iNAT internal control for influenza A failed in one sample, and the Sofia internal controls for influenza A and B failed in four samples. These invalid results were excluded from further analysis including the one Sofia sample that was subsequently positive for influenza A by RT-PCR.

Compared to RT-PCR, Alere iNAT detected 28/36 (77·8%) influenza A positives versus 25/35 (71·4%) and 16/36 (44·4%) for Sofia (*P *=* *0·59, Fisher's exact test) and BinaxNOW (*P *=* *0·007), respectively. No significant difference in sensitivity was noted for the Alere iNAT, Sofia and BinaxNOW in detecting influenza A(H1N1)pdm09 and seasonal A/H3N2 (*P = *1·0 for each assay). For influenza B, Alere iNAT detected 9/12 (75%) of those positive by RT-PCR, versus 4/12 (33·3%) and 3/12 (25·0%) for Sofia (*P *=* *0·10) and BinaxNOW (*P *=* *0·04), respectively. Alere iNAT gave two (1·0%) false-positive influenza B results and the Sofia produced two (1·0%) false-positives for influenza A and one (0·5%) for influenza B. In addition, one sample (0·5%) was positive by Sofia for both influenza A and B but was negative for all respiratory viruses by RT-PCR. False-positive influenza results due to cross-reactivity with other respiratory viruses was not observed for any of the testing platforms. The performance characteristics for all three RIDTs are summarised in Table1.

**Table 1:** Performance statistics for Alere™ iNAT, Sofia® and BinaxNOW® using reverse-transcription polymerase chain reaction as the reference standard

	Influenza A			Influenza B		
	Alere™ iNAT	Sofia®	BinaxNOW®	Alere™ iNAT	Sofia®	BinaxNOW®
Sensitivity (%) (95% CI)	77·8 (61·7–88·5)	71·4 (54·8–83·8)	44·4 (29·5–60·4)	75·0 (46·2–91·7)	33·3 (13·6–61·2)	25·0 (8·3–53·8)
Specificity (%) (95% CI)	100 (97·3–100)	98·2 (94·5–99·6)	100 (97·3–100)	99·0 (96·0–99·9)	99·5 (97·0–99·9)	100 (97·6–100)
PPV (%) (95% CI)	100 (85·7–100)	89·3 (72·0–97·1)	100 (77·3–100)	81·8 (51·2–96·0)	66·7 (29·6–90·8)	100 (38·2–100)
NPV (%) (95% CI)	95·4 (91·0–97·8)	94·1 (89·4–97·6)	89·2 (83·9–93·0)	98·4 (95·3–99·7)	95·8 (91·9–98·0)	95·5 (91·5–97·7)

PPV, Positive predictive value; NPV, Negative predictive value.

The sensitivity of the Alere iNAT was affected by the viral load present in each sample as indirectly indicated by the RT-PCR cycle threshold (*C*_t_) values. The median *C*_t_ values for samples positive by Alere iNAT and RT-PCR were 25·28 [Interquartile range (IQR), 22·86–28·61] and 28·16 [IQR, 24·44, 33·68] for influenza A and B, respectively, versus 30·98 [IQR, 29·4, 32·54] and 28·53 [IQR, 28·16, 32·53], respectively, for samples that were positive by RT-PCR only (*P *<* *0·05 by two-tailed Mann–Whitney *U*-test for influenza A; *P *>* *0·05 for influenza B).

## Discussion

Alere iNAT has recently received FDA clearance for the detection of influenza A and B viruses.^15^ The present study evaluated the performance of Alere iNAT against existing RIDTs using RT-PCR as the reference standard for the detection of influenza viruses on clinical specimens collected from patients with an ILI. Others have previously compared Alere iNAT against RT-PCR and/or viral culture using prospectively collected or frozen samples.^6^,^7^,^16^ Overall, Alere iNAT has demonstrated superior performance to the other RIDTs, particularly in the detection of influenza B.

According to the manufacturer, the minimum possible UTM volume between 0·5 and 3 ml is recommended for clinical swab elution in order to maintain the analytical sensitivity of the Alere iNAT.^17^ In this study, nasopharyngeal swab samples were eluted in 2 ml of UTM to allow testing using three different assays. However, this may have compromised the performance of all RIDTs tested. The overall sensitivities reported herein compared to RT-PCR for the Alere iNAT of 77·8% and 75% in the detection of influenza A and B viruses, respectively, are comparable to results obtained by previous investigators using frozen respiratory samples collected in 3 ml of UTM. In one such study where RT-PCR was also used as the reference standard, the sensitivity of the Alere iNAT was 73·2% for the detection of influenza A viruses and 97·4% for the detection of influenza B.^6^

The sensitivity of diagnostic tests for influenza is also affected by sample type and the origin of the sample.^18^ All three RIDTs used in this study are only validated for testing of upper respiratory tract samples. The amount of virus present in the upper respiratory tract is generally inversely proportional to the elapsed duration between the onset of symptoms and specimen collection.^19^ Although specimens were processed within 24 hours of receipt in the laboratory in this study, information about the time of symptom onset to specimen collection was not available.

The lack of paediatric samples may have also lowered the observed sensitivity of all RIDTs tested; previous meta-analyses have suggested improved RIDT sensitivity of up to 13% in children as they generally have higher viral loads and prolonged viral shedding compared to adults.^3^,^20^ A recent study of Alere iNAT performance in children and adolescents reported sensitivities and specificities of 88·8% and 98·3%, respectively, for influenza A and 100% and 100%, respectively, for influenza B compared to RT-PCR.^16^

Of note, the sensitivity of Sofia and BinaxNOW in detecting influenza B virus was lower than that observed for the Alere iNAT and lower than their respective sensitivities in the detection of influenza A. Previous evaluations using clinical samples or virus culture supernatants containing human isolates of influenza B virus have also showed varying sensitivities amongst the different RIDTs in detecting this virus.^20^,^21^

Similar to previous reports, several false-positive Alere iNAT and Sofia results were observed in this study, which in the clinical setting may have led the prescription of inappropriate antiviral therapy or delays in obtaining the correct diagnosis.^16^,^22^ The cause of these false-positive results is unclear, but may be due to cross-reactivity with another pathogen that was not covered in our RT-PCR assay, or manufacturing defects of test cartridges or specimen receivers.

As RIDTs only detect influenza viruses, other respiratory viruses that may be cocirculating at the time that can cause ILIs may be missed, as evidenced in the present study.^23^ Although influenza treatment is the most commonly available and prescribed antiviral for ILIs at present, we anticipate that this will change given the number of antivirals that are currently in phase II or III trials.^24^ In the future, the diagnosis of a definitive aetiological agent responsible for the ILI will be needed to facilitate prescription of targeted antiviral therapy.

In conclusion, the sensitivity of Alere iNAT was at least equivalent to that of Sofia and was superior to that of the BinaxNOW ICT in the detection of influenza A and B. Although this study was conducted within a virology reference laboratory, Alere iNAT can be used by trained personnel in a variety of healthcare settings given its semi-automated nature. Unlike Sofia, however, direct data transfer between sites is not available with the current Alere iNAT platform. Features that support the use of Alere iNAT as a true point-of-care test include the availability of results within 15 minutes of specimen receipt and quality control systems including an objective reader and internal control in each test cartridge. However, caution needs to be applied when interpreting negative tests where influenza is still strongly suspected.
